# STAT3 governs the HIF-1α response in IL-15 primed human NK cells

**DOI:** 10.1038/s41598-021-84916-0

**Published:** 2021-03-29

**Authors:** Anna Coulibaly, Sonia Y. Velásquez, Nina Kassner, Jutta Schulte, Maria Vittoria Barbarossa, Holger A. Lindner

**Affiliations:** 1 Department of Anesthesiology and Surgical Intensive Care Medicine, University Medical Center Mannheim, Mannheim Institute for Innate Immunoscience (MI3), Medical Faculty Mannheim, Heidelberg University, 68167 Mannheim, Germany; 2 Interdisciplinary Center for Scientific Computing, Heidelberg University, 69120 Heidelberg, Germany; 3 Frankfurt Institute of Advanced Studies, 60438 Frankfurt, Germany

**Keywords:** Biochemistry, Cell biology, Immunology

## Abstract

Natural killer (NK) cells mediate innate host defense against microbial infection and cancer. Hypoxia and low glucose are characteristic for these tissue lesions but do not affect early interferon (IFN) γ and CC chemokine release by interleukin 15 (IL-15) primed human NK cells in vitro. Hypoxia inducible factor 1α (HIF-1α) mediates cellular adaption to hypoxia. Its production is supported by mechanistic target of rapamycin complex 1 (mTORC1) and signal transducer and activator of transcription 3 (STAT3). We used chemical inhibition to probe the importance of mTORC1 and STAT3 for the hypoxia response and of STAT3 for the cytokine response in isolated and IL-15 primed human NK cells. Cellular responses were assayed by magnetic bead array, RT-PCR, western blotting, flow cytometry, and metabolic flux analysis. STAT3 but not mTORC1 activation was essential for HIF-1α accumulation, glycolysis, and oxygen consumption. In both primed normoxic and hypoxic NK cells, STAT3 inhibition reduced the secretion of CCL3, CCL4 and CCL5, and it interfered with IL-12/IL-18 stimulated IFNγ production, but it did not affect cytotoxic granule degranulation up on target cell contact. We conclude that IL-15 priming promotes the HIF-1α dependent hypoxia response and the early cytokine response in NK cells predominantly through STAT3 signaling.

## Introduction

Natural killer (NK) cells exert cytotoxicity against infected and transformed cells^1^ and contribute to the regulation of immune responses through the production of cytokines^2^. These include, foremost, interferon (IFN) γ that supports antigen presentation^3,4^ and the CC chemokines CCL3, CCL4 and CCL5 that attract further immune cells^5,6^. In as little as 4─16 h, NK cells migrate to sites of infection^7–9^ and cellular transformation^10^. These inflammatory lesions are frequently characterized by local hypoxia^1,11,12^. The transcription factor hypoxia inducible factor 1α (HIF-1α) is the master regulator of cellular adaption to hypoxia. HIF-1α is constitutively produced and undergoes prolyl hydroxylation in the presence of oxygen resulting in its rapid degradation by the ubiquitin–proteasome system. Under hypoxia, HIF-1α is stabilized, translocates to the nucleus, and activates transcription of hypoxia-responsive genes including glycolytic genes^13^. Different signaling pathways have been reported to enhance HIF-1α production in various types of immune cells in an oxygen independent manner, supporting their metabolic and thus functional adaption to inflammatory conditions. These pathways include signaling through mechanistic target of rapamycin (mTOR) in macrophages^14^, neutrophils^15^, basophils^16^, and T lymphocytes^17,18^, and through nuclear factor-κB (NF-kB) in myeloid cells^19^ and B lymphocytes^20^. In addition, signal transducer and activator of transcription 3 (STAT3) mediates HIF-1α production in activated T lymphocytes^21^ and B lymphocytes^20^. We recently presented experimental evidence for a role of mTOR complex 1 (mTORC1) and STAT3 in HIF-1α activation in response to the cytokine interleukin 15 (IL-15) in human NK cells cultured under chemical hypoxia and developed a mathematical model of this process^22^.

IL-15 is produced by monocytes, macrophages, and dendritic cells, promotes the survival of NK cells, and rapidly improves their effector functions during an immune response by initiating Janus kinase 1 (JAK1)/STAT3 and JAK3/STAT5 signaling^23–25^. STAT5 mainly serves NK cell homoeostasis by promoting survival, maturation and proliferation, while STAT3 plays a yet controversial role in the regulation of NK cell cytotoxicity^26^. For instance, STAT3 represses the expression of the cytotoxic perforin and granzyme B genes in mouse^27^ and mediates the downregulation of cell surface expression of the two activating human NK cell receptors NKG2D and NKp30 by tumor derived IL-6 and IL-8^28^. In apparent contrast to these inhibitory functions, STAT3 was previously shown to drive the expression of the *NKG2D* gene^29^.

In addition to JAK/STAT signaling, high dose IL-15 induces mTORC1 activity in NK cells, a signaling axis that sustains their expansion in the bone marrow^30^. Overnight stimulation of mature NK cells with IL-15, that is well beyond the priming phase, is known to cause mTORC1 dependent metabolic switching to oxidative phosphorylation and glycolysis that their effector functions then rely on^31–33^. However, little is known about the short-term immunometabolic regulation of NK effector functions. Contrary, to the long-term metabolic requirements^31–33^, we recently found IL-12/IL-18 stimulated short-term release of IFNγ and CCL3, CCL4, and CCL5 from both normoxic and hypoxic IL-15 primed human NK cells to be essentially independent of glucose availability^34^. This has questioned the importance of glycolysis as a cellular source of energy and anabolic precursors for the early cytokine response in human NK cells even under hypoxia. Yet, glucose deprivation for 4 h still reduced intracellular IFNγ abundance by around 30%^34^ which agrees with long-term dependence of IFNγ release on glycolysis^31–33^.

Several clinical trials using recombinant human IL-15 are registered with the National Cancer Institute (https://clinicaltrials.gov/). Administration of recombinant human IL-15 to patients with metastatic disease was safe and caused efflux of NK cells from the circulation within 30 min followed by massive hyperproliferation by 48 h and return to baseline up on cessation of IL-15 administration^35^.

In this study, we sought further insight into the importance of mTORC1 and STAT3 signaling for the early hypoxia response in IL-15 primed human NK cells. Because genetic manipulation of primary NK cells is technically challenging^36^, we used chemical inhibition of mTORC1 activity and STAT3 phosphorylation to this end. STAT3 but not mTORC1 was essential for HIF-1α protein accumulation and glycolysis. STAT3 inhibition also prevented priming induced secretion of CCL3 and CCL4, and partially reduced secretion of CCL5, and it strongly reduced cellular production of IFNγ. Cytotoxic granule degranulation, by contrast, was not affected. We conclude that IL-15 priming conditions NK cells for hypoxia through a STAT3-HIF-1α signaling axis and that STAT3, additionally, supports the early cytokine response. In the context of IL-15 immunotherapy, pharmacological targeting of STAT3 may thus be preferably only some time following the localization and priming-enhanced adaption of NK cells to hypoxic sites such as the tumor microenvironment.

## Materials and methods

This research involved human blood samples and was conducted in accordance with the World Medical Association Declaration of Helsinki (https://www.wma.net/policies-post/wma-declaration-of-helsinki-ethical-principles-for-medical-research-involving-human-subjects/). We have described previously the procedures for the isolation and culture of human NK cells, flow cytometry, bead array analysis, RT-PCR and extracellular flux analysis^34,37^ as well as western blotting^22^. In the following, a brief outline is given, and the specific reagents used here are identified.

### Cell isolation and culture

Blood from healthy volunteers was sampled with their informed consent and under medical supervision at the University Medical Center Mannheim. Donors at the local Red Cross Blood Donor Service provided informed consent to the use of residual blood constituents including buffy coats for research as part of the standard blood donation enrolment. NK cells were isolated (NK-Cell Isolation Kit, Miltenyi Biotec) from whole blood of healthy volunteers for extracellular flux analysis and from buffy coats obtained through the local Red Cross Blood Donor Service for all other experiments. Cell viabilities by trypan blue staining were ≥ 98% (Countess, Invitrogen). The purity of NK cells was determined by flow cytometry as described^37^ and preparations with a phenotype of ≥ 95% CD56^+^CD3^−^ and ≤ 1% each CD3^+^, CD14^+^, CD15^+^, and CD19^+^ were judged as pure and were further cultured. Cells were plated at 10^6^/mL in RPMI 1640 medium supplemented with 10% fetal bovine serum and 2 mM L-glutamine. Cells were maintained in a standard tissue culture incubator at 37 °C and 5% CO_2_ (normoxia) or in an oxygen-controlled Galaxy 48R CO_2_ incubator (New Brunswick) with a nitrogen gas line to establish 1% O_2_ at 37 °C and 5% CO_2_ (hypoxia).

### Cell treatment

Recombinant human cytokines IL-12 and IL-15 were obtained from PeproTech and IL-18 from MBL International. They were used for high-dose treatments at final culture concentrations of 10, 45 and 50 ng/mL, respectively. NK cells were cultured under normoxia or hypoxia for 16 h and primed with IL-15 for further 6 h in the presence or absence of indicated inhibitors. The mTORC1 inhibitor rapamycin (Merck Millipore) was used at 25 nM, the proteasome inhibitor MG132 (Sigma-Aldrich) at 5 μM, the translation elongation inhibitor cycloheximide (Sigma-Aldrich) at 100 μM and the STAT3 inhibitors S3I-201 (referred to as S3I within the figures) and stattic (CAS 19983–44-9) (Merck Millipore) at 200 µM and 5 µM, respectively. Dimethyloxalylglycine (DMOG) (Selleck Chemicals) was used at 20 µM to establish chemical hypoxia (Supplementary Fig. S1). Dimethyl sulfoxide (DMSO) (Sigma-Aldrich) served as vehicle control.

### Flow cytometry

Fluorochrome-conjugated monoclonal antibodies (mAbs) were purchased from BD Biosciences (IFNγ-APC and mouse IgG1κ-APC [MOPC-21] as isotype control, Stat3 (pS727)-BV421 [49/p-Stat3] and mouse IgG1κ-BV421 [X40] as isotype control). For the assessment of glucose transporter 1 (GLUT1) surface expression, NK cells were stained with Glut-1.RBD (GFP) (PeloBiotech) for 20 min at 37 °C. For intracellular staining of IFNγ and pSTAT3, isolated NK cells were fixed and permeabilized using the BD Cytofix/Cytoperm Fixation/Permeabilization Kit and BD Cytofix and Perm/Wash Buffer III (BD Biosciences), respectively, following the manufacturer’s recommendations.

To assess NK cell degranulation upon target cell contact, 2–5 × 10^6^ K-562 leukemic target cells were stained with CytoTell Green stock solution (500X) (AAT Bioquest) at a final concentration of 0.25X for 15 min at 37 °C. Cells were washed once with PBS, pelleted and resuspended in RPMI 1640 medium (10% FBS, 2 mM L-glutamine) 24 h before the experiment. For the degranulation assay, 10^6^ NK cells were co-incubated with 10^5^ CytoTell Green stained K-562 cells for 4 h. Anti-CD107a-APC [Clone H4A3] mAb, mouse IgG1 κ-APC [Clone MOPC-21] as isotype control and monensin (BD Biosciences) were added at the start of the assay. IL-15 was present during the 4-h co-incubation in all experiments and, additionally, rapamycin and S3I-201 as indicated. At the end, the co-incubated cells were stained for NK cell surface markers (CD3-PerCP, CD56-PE-Cy7) for 15 min, washed once and resuspended with Cell Wash (BD Biosciences). Cells were then stained with SYTOX Blue Dead Cell Stain (Life Technologies) at 0.4 nM for 5 min before data acquisition.

A total of 10^5^ isolated stained NK cells were acquired on a FACSLyric cytometer using BD FACSuit version 1.2.1 (BD Biosciences) and analyzed using FlowJo version 10.4.1 software (Tree Star). We gated subsequently on singlets in the forward scatter area versus height plot and on lymphocytes in the sideward scatter area versus forward scatter area plot as described^34^. For the quantitation, of GLUT1, IFNγ and pSTAT3^Ser727^, respective proportions of stained cells and median fluorescence intensity (MFI) values were identified from fluorescence intensity histograms. Apoptosis following cell treatment with rapamycin, S3I-201 and stattic was detected by annexin V-APC and 7-amino-actinomycin D (7-AAD) staining (BD Biosciences). To estimate frequencies of CD107a^+^ NK cells, we also gated on singlets, lymphocytes and then the CytoTell Green^-^ and SytoxBlue^-^ fraction prior to the assessing the CD107a^+^ fraction of CD3^-^CD56^+^ NK cells.

### Bead array analysis

MILLIPLEX MAP kits were used to detect pRPS6^Ser235^^/236^ (48-611MAG) and pSTAT3^Ser727^ (48-680MAG) and secreted CCL3, CCL4, and CCL5 (CCL3/4/5) (HCYTOMAK-60 K) according to the manufacturer's protocols (Merck Millipore). The plates were read on a MAGPIX system (Luminex). Duplicate determinations were averaged using MILLIPLEX Analyst software. The results are presented as background corrected MFI values for pRPS6^Ser235/236^ and pSTAT3^Ser727^ and as absolute concentrations for CCL3/4/5.

### Western blotting

Total cell extracts were prepared by lysing the cells for 15 min in NP-40 buffer (50 mM Tris–HCl, pH 7.5, 120 mM NaCl, 20 mM NaF, 1 mM EDTA, 6 mM EGTA, 15 mM sodium pyrophosphate, 1 mM PMSF, 0.1% Nonident P-40) followed by centrifugation for 20 min at 14,000 × g. Cleared lysates were analyzed directly by SDS-PAGE and western blotting. Proteins were visualized using Enhanced Chemiluminescent solution (Thermo Fisher) and a FUSION Vilber imager. Anti-HIF-1α (#2185), anti-TPI (ab96696), anti-PGK1 (ab38007), and anti-PDK1 (EPR19573, ab207450) were obtained from Abcam. β-Actin was detected as loading control (8H10D10, Cell Signaling Technology). Raw images of the complete blots for the experiments shown are provided as Supplementary information.

### RT-PCR

Total RNA was subjected to TaqMan RT-PCR gene expression analysis on a 7900HT Fast Real Time PCR instrument (Applied Biosystems) as described^37^. Assay IDs are assembled in Supplementary Table S1.

### Extracellular flux analysis

Extracellular acidification rate (ECAR) and oxygen consumption rate (OCR) were measured on a Seahorse XFp analyzer (Agilent Technologies) using the Seahorse XFp glycolysis stress kit. For comparison of three culture conditions, cells were plated in duplicate into miniplates at 2 × 10^5^ cells/well. During flux measurements, cells were kept in the absence or presence of IL-15 and inhibitors at the same concentrations as during the respective preceding 6-h cell treatment. For measurements under hypoxia, the XFp analyzer was placed under an oxygen- and temperature-controlled hood as described^34^. Data was evaluated using Seahorse Wave software (Agilent Technologies).

### Data representation and statistical analysis

Data is presented as bar charts with mean values and standard deviations (SD) and overlaid scatter plots with a color scheme to identify data points from independent experiments with NK cells from different donors and, thereby, to allow identifying the trend within each experiment. Numbers of independent experiments (n) are given in the figure legends. For western blots, one out of three independent experiments is shown. We used GraphPad Prism software V7.04 for statistical analyses. Differences between experimental groups were evaluated using the Friedman test with Dunn’s test for post-hoc pairwise comparisons or Wilcoxon signed-rank test as indicated. The *p* values < 0.05 were considered statistically significant.

### Ethics approval and consent to participate

Ethical approval was granted by the Ethics Committee Medical Faculty Mannheim (approval number 2016-521N-MA).

## Results

### IL-15 priming induces HIF-1α production in human NK cells only partially through mTORC1

**Figure 1:**
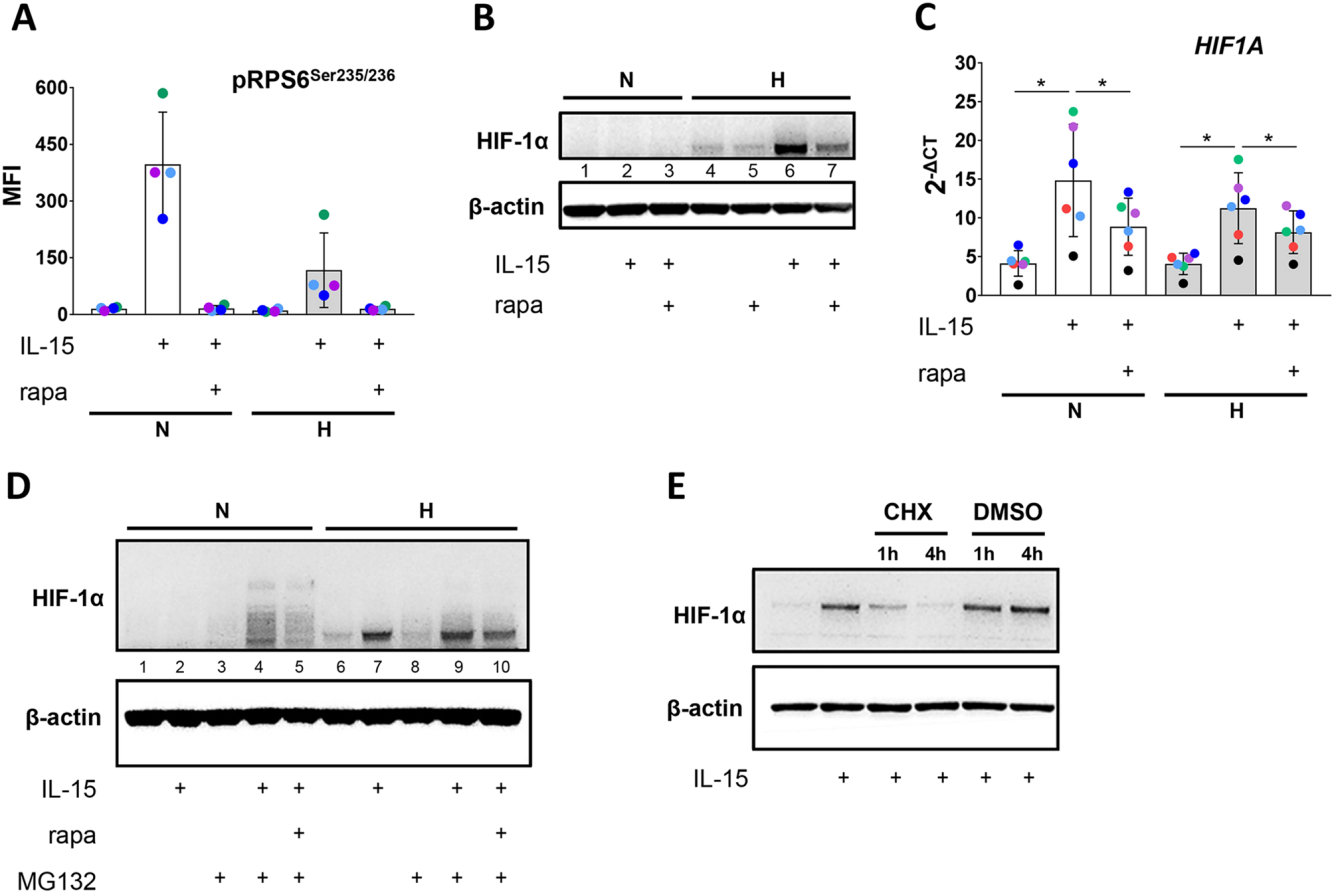
IL-15 priming induces HIF-1α production only partially through mTORC1. (**A**–**D**) NK cells were incubated under normoxia (N, 21% O_2_) or hypoxia (H, 1% O_2_) for 16 h, primed with IL-15 for additional 6 h in the presence or absence of rapamycin (rapa) and the proteasome inhibitor MG132 as indicated. (**A**) Bead array analysis of pRPS6^Ser235^^/236^. Median fluorescence intensities (MFI) are represented as mean values ± SD (n = 4). Statistical significance was not reached when applying the Wilcoxon signed-rank test. (**B**) Immunoblotting with antibodies against HIF-1α and β-actin (loading control). The blot reflects one representative experiment of three performed. (**C**) RT-PCR analysis. Linearized quantities (2^−ΔCt^) are represented as mean values ± SD (n = 6). *p < 0.05, Wilcoxon signed-rank test. (**D**) Anti-HIF-1α and anti-β-actin immunoblot analysis. The blot reflects one representative experiment of two performed. (**E**) NK cells were incubated for 4 h with DMOG (chemical hypoxia) and with or without IL-15. Where indicated, cytokine-treated cells were cultured for additional time periods with cycloheximide (CHX) or vehicle (DMSO). Cultures were subjected to anti-HIF-1α and anti-β-actin immunoblot analysis. The blot reflects one representative experiment of three performed.

We previously reported that culturing of human NK cells under hypoxia for 22 h and priming with IL-15 during the final 6 h synergistically enhanced expression of HIF-1α target genes including glycolytic genes^37^. Because of its known role for long-term NK cell metabolic switching^33^ we first analyzed the importance of mTORC1 for the accumulation of HIF-1α, a major inducer of glycolysis, in response to IL-15 priming using rapamycin, an inhibitor of mTORC1 kinase activity, at a non-toxic concentration (Supplementary Fig. S2). We confirmed that rapamycin prevented priming dependent phosphorylation of the mTORC1 downstream target ribosomal protein S6 (pRPS6^Ser235/236^) (Fig. 1A).

Next, we assessed HIF-1α levels under hypoxia, priming, and mTORC1 inhibition. As expected, HIF-1α was only detectable under hypoxia (Fig. 1B). Its levels were hardly affected by rapamycin (lane 5 vs. 4) and were further increased by priming without rapamycin (lane 6 vs. 4) and with rapamycin (lane 7 vs. 4). The observed IL-15/hypoxia synergy in elevating HIF-1α protein levels may be explained by enhanced *HIF1A* gene transcription, and subsequent protein translation, combined with hypoxia dependent HIF-1α stabilization. Indeed, IL-15 priming increased *HIF1A* gene expression on average fourfold under normoxia and threefold under hypoxia (Fig. 1C). This increase was reduced by rapamycin indicating a role for mTORC1 in the transcriptional regulation of *HIF1A*, possibly resulting in also reduced protein levels through rapamycin following hypoxic priming (Fig. 1B, lane 7 vs. 6).

Increased *HIF1A* gene expression through IL-15 priming translated into elevated HIF-1α protein levels only under hypoxia because its oxygen dependent proteasomal degradation very likely abrogated any priming effect on HIF-1α levels under normoxia. Prevention of HIF-1α degradation through chemical proteasome inhibition by MG132 during priming (Fig. 1D) indeed resulted in accumulation of higher-molecular-weight forms, most probably polyubiquitin-chain conjugated forms of HIF-1α (cf.^38^), in normoxia (lane 4). Rapamycin showed stronger effects under normoxia (lane 5 vs. 4) than hypoxia (lane 10 vs. 9).

HIF-1α accumulation through hypoxic IL-15 priming may be limited through a proteasome independent decrease of the protein. We assessed the velocity for this type of decay using chemical hypoxia established by the chemical pan-prolyl hydroxylase inhibitor DMOG. NK cells were exposed to chemical hypoxia and IL-15 for 4 h before general protein biosynthesis was blocked by adding cycloheximide. While priming-enhanced HIF-1α levels remained stable in the presence of DMSO vehicle, they were reduced in the presence of cycloheximide after 1 h and were barely detectable after 4 h (Fig. 1E).

The ability to rapidly amplify HIF-1α levels under hypoxia is not unique to IL-15. We found that a 4-h exposure of hypoxic human NK cells to chemical hypoxia and IL-12 in combination with IL-18 had a similar effect as IL-15 priming (Supplementary Fig. S3). Together, this data (Fig. 1 and Supplementary Fig. S3) indicates that HIF-1α accumulation in NK cells is cytokine-regulated and requires continuous protein synthesis.

### IL-15 priming stimulated glycolysis in hypoxic NK cells partially depends on mTORC1

**Figure 2:**
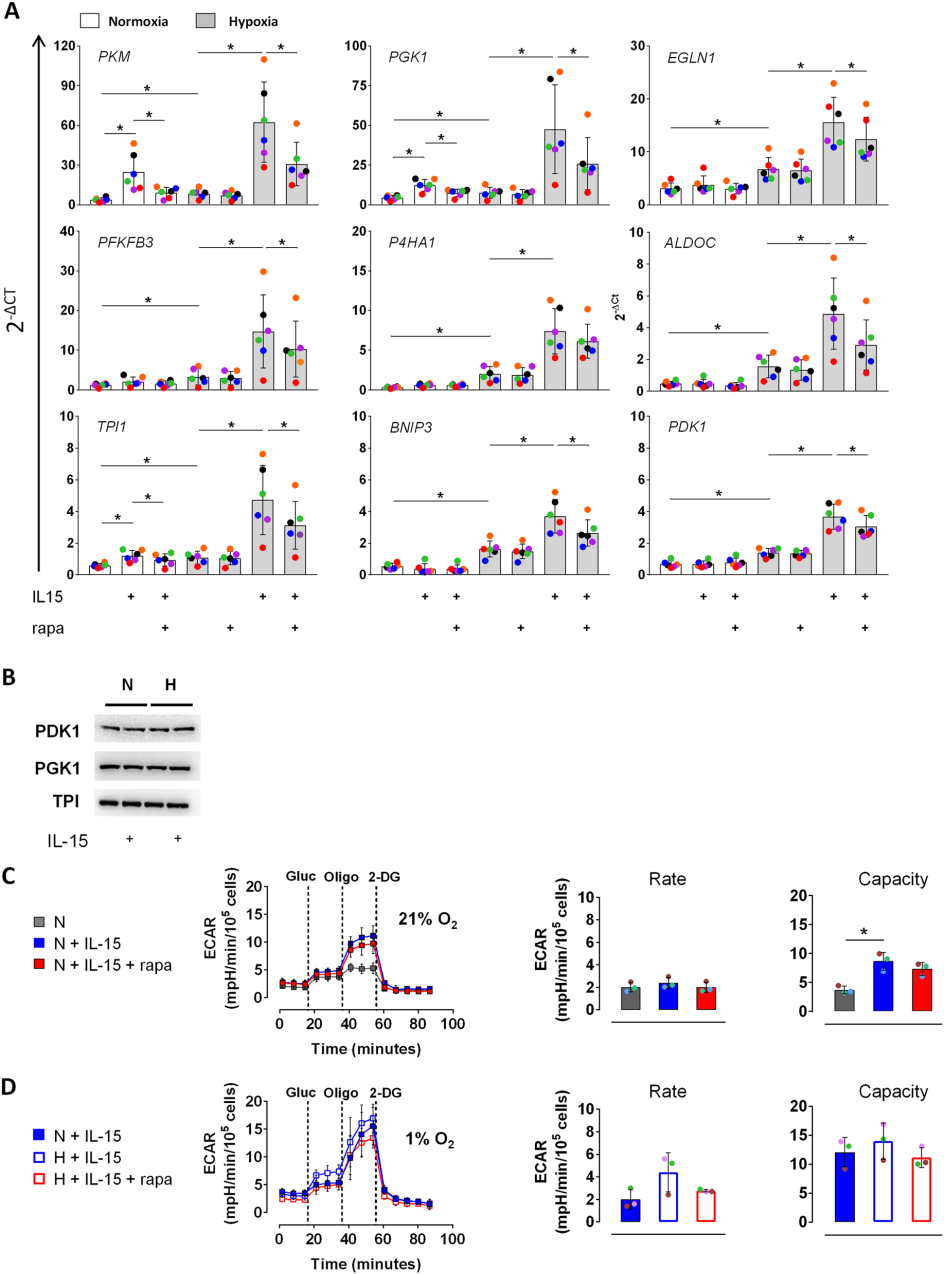
The glycolytic response of NK cells to priming and hypoxia partially depends on mTORC1. (**A**–**D**) NK cells incubated under normoxia (N, 21% O_2_) or hypoxia (H, 1% O_2_) for 16 h were primed with IL-15 for additional 6 h in the presence or absence of rapamycin (rapa) as indicated. (**A**) Expression analysis of hypoxia-regulated genes by RT-PCR. Mean values ± SD for linearized quantities (2^−ΔCt^) are shown (n = 6). *p < 0.05, Wilcoxon signed-rank test. (**B**) Immunoblotting with antibodies against PDK1, PGK1 and TPI. Aliquots of the same samples were analyzed as in Fig. 1d, lanes 1, 2, 6 and 7. The blot reflects one representative experiment of three performed. (**C**) and (**D**) Glycolytic parameters. Extracellular acidification rate (ECAR) values were measured over time under atmospheric oxygen, i.e., 21% O_2_ (**C**) or in a hypoxic chamber at 1% O_2_ (**D**). Each data point represents an average ± SD of three independent experiments. Dashed lines indicate additions of glucose (Gluc), oligomycin (Oligo), and 2-deoxyglucose (2-DG). *p < 0.05, Friedman test with Dunn’s test for post-hoc pairwise comparisons.

One of the major cellular responses elicited by HIF-1α is glycolysis^39^. Given that mTORC1 only partially contributes to HIF-1α production (Fig. 1), we next assessed its actual significance for glycolysis following normoxic and hypoxic IL-15 priming. Profiling of HIF-1α target genes confirmed synergistic up-regulation by priming and hypoxia for the glycolytic genes *PKM*, *PGK1*, *PFKFB3*, *ALDOC*, *TPI1* and *PDK1*, and additionally for *EGLN1*, *PAHA1*, and *BNIP3* (Fig. 2A) (cf.^37^). Mean synergistic IL-15/hypoxia increases ranged from sixfold for *PDK1* to 17-fold for *PKM*. In the presence of rapamycin, this synergy was blunted but persisted.

Western blot detection of phosphoglycerate kinase 1 (PGK1), triose-phosphate isomerase (TPI1), and pyruvate dehydrogenase kinase isozyme 1 (PDK1) did not reveal any protein level changes through priming, hypoxia, and their combination (Fig. 2B).

Next, we measured the influence of mTORC1 inhibition on glycolytic parameters. Under normoxia, IL-15 priming and rapamycin hardly affected glycolytic rate, but priming enhanced glycolytic capacity twofold with and without rapamycin (Fig. 2C). Additionally, glycolytic parameters of normoxically and hypoxically primed cells were measured together in a hypoxic chamber (Fig. 2D). In this setting, hypoxic compared to normoxic pre-culture doubled glycolytic rate which appeared to be prevented by rapamycin. Glycolytic capacity, however, was again unaffected by the mTORC1 inhibitor indicating a role for an additional signaling pathway in supporting the early glycolytic response to hypoxia in primed human NK cells.

### IL-15 priming induced HIF-1α accumulation and glycolysis in NK cells depend on STAT3

**Figure 3:**
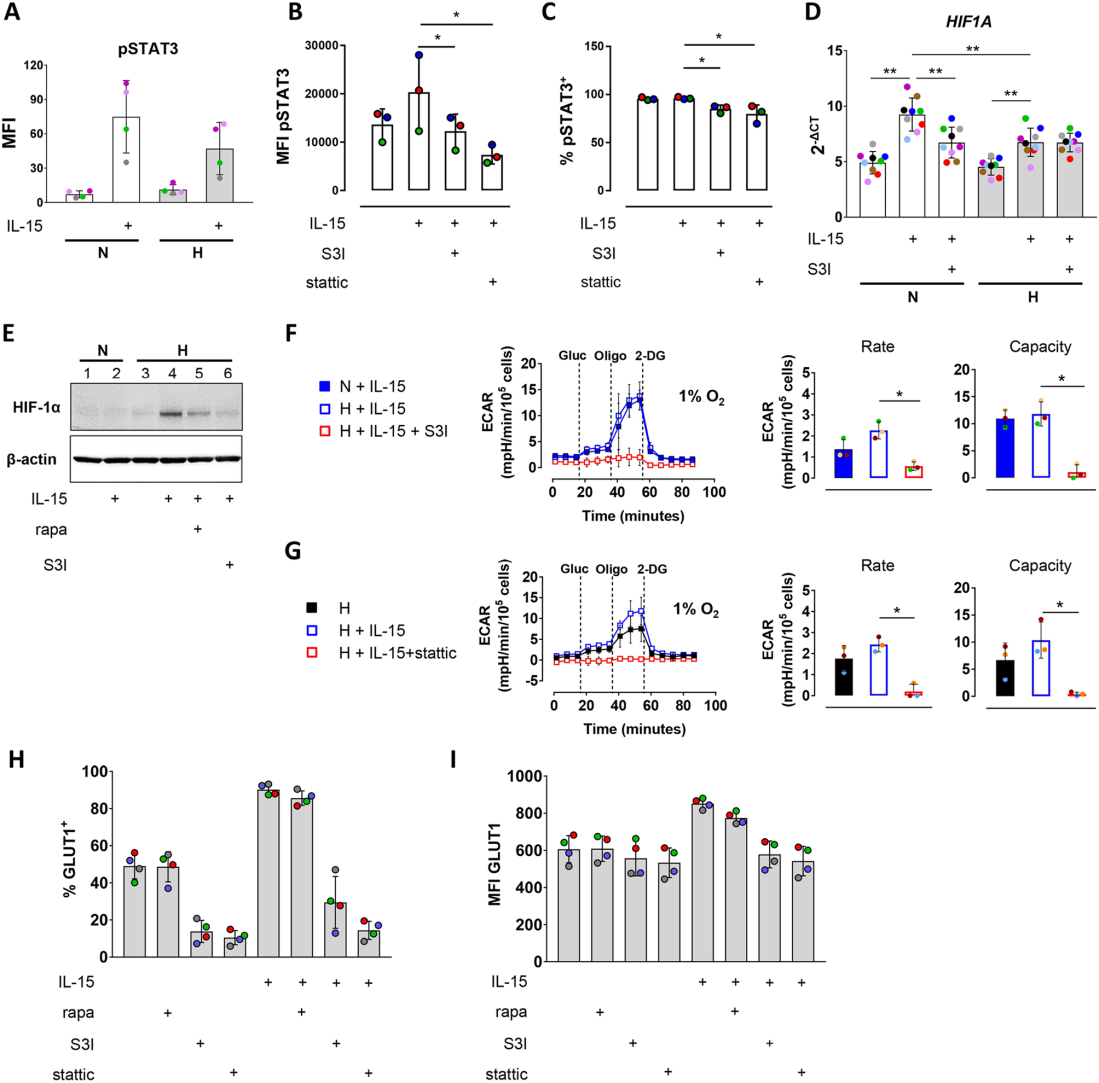
IL-15 priming induced HIF-1α accumulation and glycolysis in NK cells depend on STAT3. (**A**–**I**) NK cells were incubated under normoxia (N) or hypoxia (H) for 16 h and primed with IL-15 for additional 6 h in the presence or absence of STAT3 inhibitors (S3I-201, stattic) or rapamycin (rapa) as indicated. (**A**) Bead array analysis of pSTAT3^Ser727^. Mean values ± SD of median fluorescence intensities (MFI) are shown (n = 4). The level of statistical significance was not reached when applying the Wilcoxon signed-rank test. (**B**) and (**C**) Flow cytometric data for the proportions of (**B**) pSTAT3^Ser727^ MFI and (**C**) the proportion of pSTAT3^+^ NK cells, shown as mean values ± SD (n = 3). *p < 0.05, Friedman test with Dunn’s test for post-hoc pairwise comparisons. (**D**) *HIF1A* gene expression analysis by RT-PCR. Linearized quantities (2^−ΔCt^) are represented as mean values ± SD (n = 9). **p < 0.01, Wilcoxon signed-rank test. (**E**) Immunoblot analysis with antibodies against HIF-1α and β-actin. The blot reflects one representative experiment of three performed. (**F**) and (**G**) Glycolytic parameters. Extracellular acidification rate (ECAR) values were measured over time in a hypoxic chamber at 1% O_2_. Each data point represents an average ± SD of three independent experiments. Dashed lines indicate additions of glucose (Gluc), oligomycin (Oligo), and 2-deoxyglucose (2-DG). *p < 0.05, Friedman test with Dunn’s test for post-hoc pairwise comparisons. (**H**) and (**I**) Flow cytometric data for (**H**) the proportion of GLUT1^+^ hypoxic NK cells and (**I**) GLUT1 MFI is represented as mean values ± SD (n = 4). The level of statistical significance was not reached when applying the Wilcoxon signed-rank test.

In activated B cells, STAT3-mediated *HIF1A* expression was shown to depend on serine 727 phosphorylation^20^. We therefore assessed this specific posttranslational modification in IL-15 primed human NK cells using magnetic bead array. IL-15 priming indeed enhanced mean average levels of pSTAT3^Ser727^ in human NK cells almost 11-fold under normoxia and sevenfold under hypoxia (Fig. 3A).

We used two inhibitors of STAT3 activity, S3I-201 and in some experiments additionally stattic, to probe the importance of STAT3 as a regulator of the hypoxia response. S3I-201 and stattic were able to reduce STAT3 phosphorylation on Ser727 to the level of unstimulated cells and below, respectively, (Fig. 3B and Supplementary Fig. S4). They slightly but significantly also reduced proportions of cells positive for this modification that already totaled around 95% without priming (Fig. 3C) at concentrations that had no impact on cell viability (Supplementary Fig. S5).

First, we analyzed the effect of S3I-201 on priming induced HIF-1α expression at the transcriptional and translational levels. Similarly as before (Fig. 1C), mean increases in *HIF1A* mRNA by IL-15 priming under normoxia were higher than under hypoxia (Fig. 3D). And similar to rapamycin (Fig. 1c), S3I-201 reduced priming-induced *HIF1A* upregulation under normoxia by 27%. Contrary to rapamycin, S3I-201 did however not further diminish *HIF1A* expression under hypoxia (Fig. 3D). Conversely, while rapamycin as before (Fig. 1B) only moderately reduced HIF-1α protein levels under hypoxic priming (Fig. 3E, lane 5 vs. lane 4), S3I-201 reduced them (lane 6 vs. lane 4) to hypoxic background levels (cf. lane 3).

We next considered the effect of inhibiting STAT3 on glycolysis in hypoxic primed NK cells. In contrast to rapamycin (Fig. 2D), both S3I-201 (Fig. 3F) and stattic (Fig. 3G) virtually abolished glycolytic flux. Notably, still noticeable oxygen consumption rates measured in parallel under hypoxia were reduced about two-fold at baseline by S3I-201 and almost paralyzed by stattic while rapamycin had only a minor effect (Supplementary Fig. S6).

In the absence of protein level changes for glycolytic enzymes under these conditions (Fig. 2B), we evaluated cell surface expression of GLUT1 with and without mTORC1 and STAT3 inhibition during hypoxic priming. While rapamycin had no effect, S3I-201 and stattic both reduced percentages of GLUT1 positive cells around three-fold with and without priming (Fig. 3H) and prevented the priming induced elevation of cell surface levels of GLUT1 (Fig. 3I).

### STAT3 inhibition affects the early cytokine response but not target cell induced degranulation in NK cells

**Figure 4:**
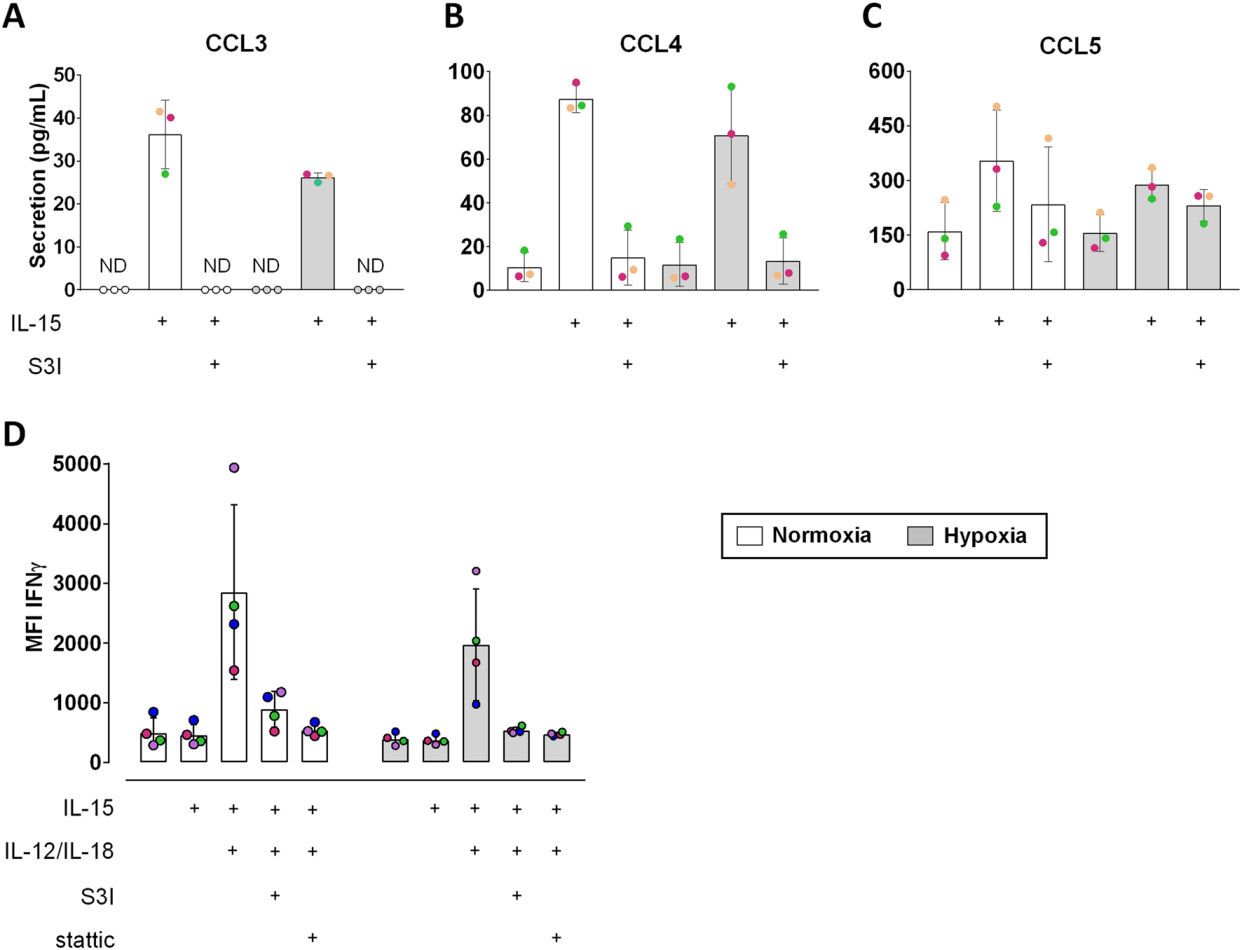
STAT3 inhibition interferes with CCL3/CCL4 secretion and IFNγ production in NK cells. NK cells were incubated under normoxia or hypoxia for 16 h and treated with IL-15 alone (**A − C**) or in combination with IL-12/IL-18 (**D**) for additional 6 h in the presence or absence of STAT3 inhibitors (S3I-201, stattic) as indicated. (**A − C**) Bead array analysis of secreted chemokines. Bars represent mean chemokine concentrations ± SD (n = 3). The level of statistical significance was not reached when applying the Wilcoxon signed-rank test. ND: not detectable. (**D**) Pooled flow cytometric data for the proportions of IFNγ median fluorescence intensities (MFI), shown as mean values ± SD (n = 4). The level of statistical significance was not reached when applying the Wilcoxon signed-rank test.

The importance of STAT3 for the cytokine response in primed NK cells is not yet known. With S3I-201 present during priming, already low levels of CCL3 released by human NK cells were undetectable (Fig. 4A) and CCL4 release was at the levels of unprimed cells (Fig. 4B). The reduction of IL-15 dependent elevation of CCL5 levels in the presence of the STAT3 inhibitor was less pronounced but still noticeable (Fig. 4C). Hypoxia appeared to reduce priming-stimulated secretion throughout. To induce production of the major NK cytokine IFNγ, we stimulated the cells for 6 h with IL-12 and IL-18 in addition to IL-15. The presence of S3I-201 and stattic both prevented a clear increase of intracellular IFNγ abundance through this treatment which appeared again lower in hypoxic than normoxic NK cells (Fig. 4D and Supplementary Fig. S7).

**Figure 5:**
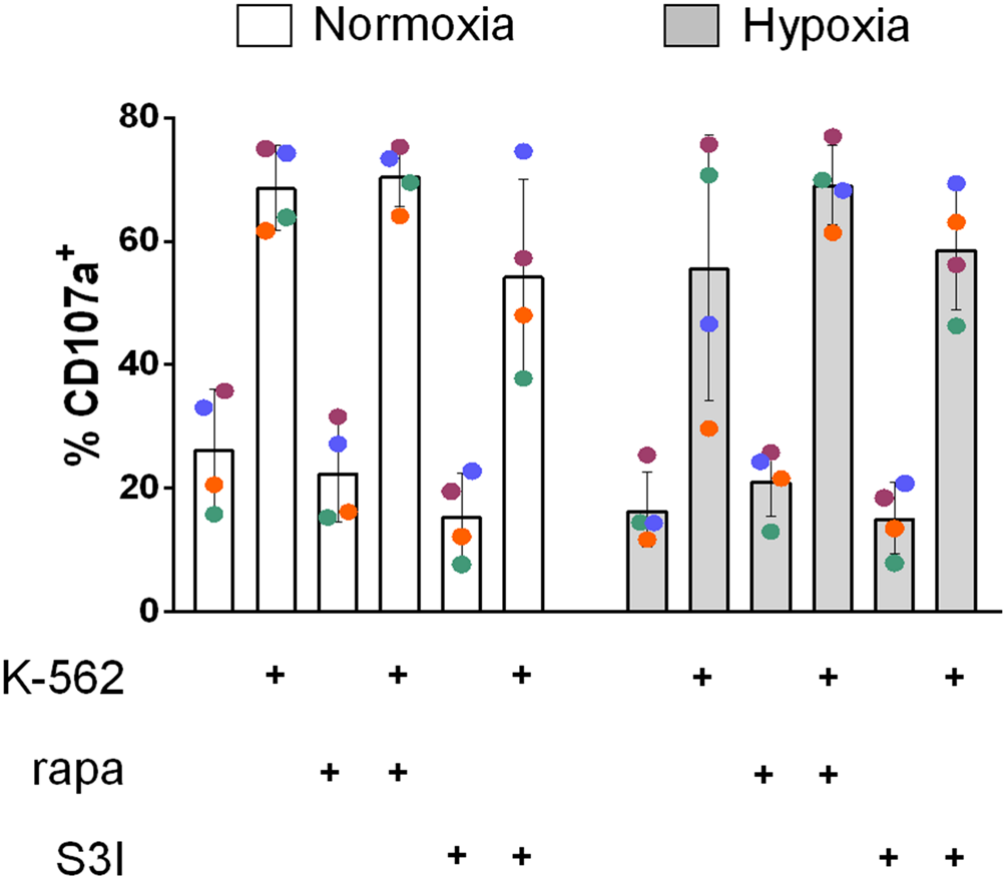
mTORC1 and STAT3 inhibition does not interfere with K-562 cell contact induced NK cell degranulation. NK cells were incubated under normoxia and hypoxia for 16 h and subsequently co-incubated with K-562 cells for 4 h in the presence of IL-15 and under continued normoxia and hypoxia, respectively, with or without rapamycin (rapa) and S3I-201 as indicated. Pooled flow cytometric data for the proportions of CD107a positive cells are shown as mean values ± SD (n = 4). The level of statistical significance was not reached when applying the Wilcoxon signed-rank test.

We have previously shown that neither normoxic nor hypoxic IL-15-priming of human NK cells for 6 h reduced subsequent killing of K-562 cells measured under normoxia^37^. Notably, tumor cells can adapt to hypoxia by activating autophagy, which has been shown to also target NK cell derived granzyme B and, thereby, protect tumor cells from NK cell mediated cytotoxicity^40,41^. This hypoxia dependent protective effect complicates interpreting direct measurement of NK cell mediated cell death of target cells following their hypoxic co-incubation. Therefore, we chose an NK cell intrinsic reporter of their cytotoxic function and assessed cell surface levels of CD107a as a proxy for degranulation of cytotoxic granules^42^ instead of measuring cell death markers in target cells. In this way, we could compare cytotoxic function of NK cells under normoxia and hypoxia (Fig. 5 and Supplementary Fig. S8). We found that the presence of neither rapamycin nor S3I-201 signifiantly altered K-562 cell induced degranulation in normoxic and hypoxic NK cells over a co-incubation period of 4 h and in the presence of IL-15. As a possible exception, S3I-201 slightly decreased CD107a surface levels in normoxic NK cells both with and without target cells.

## Discussion

Innate cytokines cooperate with hypoxia in augmenting the HIF-1α response in NK cells^22,37,43^. In human NK cells treated for 6 h with IL-15, referred to as priming, we found that predominantly STAT3 rather than mTORC1 signaling mediates this cooperation and acts, to this effect, on the level of HIF-1α protein accumulation. STAT3 activation was also essential for glycolysis and cellular respiration under hypoxia. The subordinate role of mTORC1 compared to STAT3 in the short-term HIF-1α response, in particular the metabolic response, contrasts its crucial role in switching to aerobic glycolysis up on long-term treatment (> 16 h) of NK cells with innate cytokines including IL-15^31–33,44^. The previous studies did, however, not consider the impact of hypoxia on immunometabolism and on the course of the inflammatory response. Failure to resolve inflammatory hypoxia and thus extended immune stimulation at low oxygen is known to suppress NK cell functions in vitro and in vivo, e.g., in the tumor microenvironment^45^. The original role of the HIF-1α response, however, is to promote resolution of tissue hypoxia^13^, and it is thus transient in nature^46^.

Priming increased both HIF-1α protein (Fig. 1B) and mRNA levels (Fig. 1C). Inhibition of oxygen dependent HIF-1α degradation by the proteasome inhibitor MG132 rendered polyubiquitinated HIF-1α detectable under normoxia (Fig. 1D). Without stabilization by hypoxia, the half-life of HIF-1α is very short^39^. HIF-1α levels may thus be very sensitive to changes in its protein translation rates even under hypoxia. When de novo protein synthesis was blocked by cycloheximide, detectable levels of HIF-1α protein, stabilized by chemical hypoxia, indeed dropped to background within 4 h (Fig. 1E). This indicates that the sustained biosynthesis of HIF-1α during priming is an important contributor to its accumulation in hypoxic human NK cells. Therefore, an enhancement of HIF-1α biosynthesis within this short timeframe can indeed explain the synergy between IL-15 priming and hypoxia as well as chemical hypoxia in augmenting its protein levels (Fig. 1B and^22^) and expression of its target genes (Fig. 2A) (cf.^37^).

mTORC1 plays a crucial role in long-term glycolytic switching in IL-15 treated NK cells^31–33,44^. Nevertheless, we recently showed that the mTORC1 inhibitor rapamycin blunted HIF-1α protein accumulation in human NK cells in response to IL-15 priming and chemical hypoxia by DMOG^22^. In several cancer cell lines, mTORC1 signaling supports the HIF-1α response by inhibiting HIF-1α degradation and/or promoting its production^39,47,48^. Therefore, we still considered mTORC1 a prime candidate to mediate the early HIF-1α response also in human NK cells and addressed this in more detail. We found that IL-15 priming readily induced RPS6 phosphorylation, a reporter of mTORC1 activity (Fig. 1A). Hypoxia is a known negative regulator of signaling downstream of mTORC1^49^ and indeed markedly reduced priming dependent phosphorylation of RPS6. As expected, the presence of rapamycin during priming fully prevented RPS6 phosphorylation confirming mTORC1 inhibition. Priming in the presence of rapamycin, however, only partly reduced hypoxia levels of HIF-1α and did not eliminate the IL-15/hypoxia synergy (Fig. 1B). It also had little impact on the levels of polyubiquitinated HIF-1α under normoxia with proteasome inhibition (Fig. 1D). Similarly, IL-15/hypoxia synergy in the upregulation of nine tested HIF-1α target genes was blunted but persisted except for *PKM* (Fig. 2A). Priming alone significantly increased gene expression exclusively for *PGK1*, *TPI1* and *PKM* and hypoxia alone for all of the nine tested genes. Notably, rapamycin blunted unifactorial increases clearly for priming but only marginally for hypoxia. This agrees with the concept that the partial negative effect of rapamycin on HIF-1α protein levels and target gene expression is due to a role of mTORC1 signaling in priming induced HIF-1α production, as recently modeled^22^, rather than in its degradation.

Despite the transcriptional activation of the glycolytic genes *PGK1*, *TPI1* and *PDK1* (Fig. 2A), protein levels of the encoded enzymes remained unchanged through priming and hypoxia (Fig. 2B), which equals our previous results obtained for priming and chemical hypoxia^34^. Nevertheless, priming and hypoxia both moderately increased glycolytic capacity (Fig. 2C,D) as expected^34,37^, most likely because both stimuli upregulate cell surface expression of GLUT1^34^. A negative effect through rapamycin only appeared to show through for glycolytic rate up on hypoxic priming (Fig. 2D) suggesting that mTORC1 contributes to basal glycolysis in this condition. Yet, the lack of an effect on glycolytic capacity indicates that IL-15 priming supports the early glycolytic response of human NK cells to hypoxia through an additional signaling pathway.

In this regard, the following drew our attention to STAT3 signaling as a candidate pathway. First, STAT3 is a driver of *HIF1A* mRNA expression up on immune stimulation in T lymphocytes^21^ and B lymphocytes^20^. Second, IL-15 is among a range of cytokines that rapidly activates STAT3 in mouse NK cells through canonical phosphorylation on tyrosine 705^27^. In lymphocytes, IL-15 mediated pSTAT3^Tyr705^ formation through JAK1^23^ leads to STAT3 dimerization, nuclear translocation and transcription of cell-cycle regulators, proto-oncogenes and antiapoptotic genes^50^. Non-canonical phosphorylation of STAT3 on serine 727 by mitogen activated pathway (MAP) kinase and other kinase signaling pathways, including mTORC1^48^, can further enhance STAT3 transcriptional and pro-oncogenic activities^51,52^. The requirement for serine 727 phosphorylation appears to vary with the STAT3 target gene promotor^53^. In particular, STAT3 mediated expression of *HIF1A* in activated B lymphocytes was shown to depend on serine 727 phosphorylation^20^. Therefore, we measured this modification also in IL-15 primed human NK cells. Priming indeed induced pSTAT3^Ser727^ formation under normoxia and, although to a lower extent, also under hypoxia (Fig. 3A).

The original recognition of abnormal STAT3 activation in solid tumors and its tumor promoting activity has since spurred discovery and development of numerous selective small molecule inhibitors^54,55^. Most of these interact with the phosphotyrosine (pY705) peptide binding site in the Src homology 2 (SH2) domain of the STAT3 monomer and interfere with its dimerization, tyrosine and serine phosphorylation. They include stattic, the first nonpeptidic inhibitor of STAT3^56^, and salicylic acid-based S3I-201^57^ which we both selected for this study. The presence of either compound during priming indeed fully prevented the IL-15 dependent increase in pSTAT3^Ser727^ (Fig. 3B). S3I-201, however, reduced *HIF1A* gene expression in response to priming only under normoxia and not under hypoxia (Fig. 3D). In contrast to the lack of an effect on the transcriptional response under hypoxia (Fig. 3D), S3I-201 reduced HIF-1α protein levels to hypoxic background whereas reduction by mTORC1 inhibition was, repeatedly, only partial (Fig. 1B and 3E). This data suggests that IL-15 priming synergizes with hypoxia in HIF-1α protein accumulation mainly through a STAT3 and only partially an mTORC1 mediated increase in HIF-1α biosynthesis.

In agreement with dominance of the importance of STAT3 over mTORC1 signaling in upregulating HIF-1α protein levels, S3I-201 and stattic virtually disrupted glycolysis (Fig. 3F and G) and also respiration under hypoxia (Supplementary Fig. S6) compared to little metabolic effects by rapamycin on either process (Fig. 2C,D, Supplementary Fig. S6). As for the detrimental effects of the STAT3 inhibitors on respiration at low oxygen, a role of STAT3 that reportedly depends on serine 727 phosphorylation may serve as an explanation. Namely, pSTAT3^Ser727^ can enter mitochondria and protect their integrity under conditions of oxidative stress by interacting with the electron transport chain and the mitochondrial permeability transition pore^58,59^. This role of pSTAT3^Ser727^ may not only safeguard NK cells against reactive oxidative species, e.g., produced by neutrophils to clear microbes^60^, but it is tempting to speculate that it is already crucial for mitochondrial respiration in primed NK cells.

We next considered a decrease in cell surface expression of priming and hypoxia regulated GLUT1^34^ by the STAT3 inhibitors as a likely cause of the loss in glycolytic activity by S3I-201 (Fig. 3F) and stattic (Fig. 3G). With and without priming under hypoxia, the presence of either inhibitor indeed reduced average proportions of GLUT1^+^ cells from approximately 90 and 50%, respectively, to around 10─25%, which is comparable to non-primed normoxic NK cells^34^, while rapamycin had no effect (Fig. 3H). Both STAT3 inhibitors also abolished an increase in GLUT1 cell surface abundance as judged by MFI values without an effect by mTORC1 inhibition (Fig. 3I). We propose that these changes in GLUT1^+^ in response to hypoxic priming and to STAT3 inhibition, respectively, mainly accounted for the observed increase and reduction in glycolytic flux (Fig. 3F,G).

As a limitation of our study, it has to be noted that, in addition to acting upstream of HIF-1α, STAT3 may also promote glycolysis by cooperating with HIF-1α at promoters of glycolytic genes as reported for *PDK1* in cancer cell lines^61^ or by HIF-1α-independent mechanisms that we did not address here. In this case it may further be argued that the loss in detectable HIF-1α protein upon STAT3 inhibition (Fig. 3E) resulted from virtual cessation of glycolysis (Fig. 3F,G) as a provider of amino acid precursors for protein biosynthesis. However, the ability of primed human NK cells to mount equally strong cytokine release responses to IL-12/18 stimulation with and without glucose^35^ does not support dependence of protein biosynthesis on glycolysis in this short time frame. Lastly, the proposed role of NF-kB in upregulating HIF-1α in NK cells^22^ remains to be tested experimentally.

STAT3 is known to divergently regulate the expression of cytotoxic NK effector molecules^27^ and activating receptors^28,29^, but its role in the regulation of NK cell cytokines is unclear. Although in vitro priming with IL-15 does not elicit a strong cytokine response, it moderately elevates the levels of the CC chemokines CCL3, CCL4 and CCL5 in supernatants of human NK cell cultures^37^. In addition to the regulation of HIF-1α and glycolysis, we therefore also considered the effect of STAT3 inhibition on cytokine production. We found that increasing amounts of released chemokines (CCL3 < CCL4 < CCL5) were associated with decreasing relative sensitivities of this release to STAT3 inhibition by S3I-201 (Fig. 4A─C) that may, however, stem from very similar degrees of inhibition as judged by the absolute reductions for each chemokine. Stimulation of IFNγ production, the major NK cytokine, required addition of IL-12 and IL-18 to the priming culture (Fig. 4D). Independent of oxygen availability, both STAT3 inhibitors prevented IFNγ production induced by the interleukin triple cocktail. This data demonstrates a clear effect of STAT3 on the cytokine response in primed human NK cells. Yet, the magnitude of the early cytokine response under the conditions tested was very small, and STAT3 inhibition may easily be overcome upon additional stimulation. For instance, IL-15 priming for 6 h followed by IL-12/IL-18 stimulation triggers the release of one to two orders of magnitude higher amounts of CCL3, CCL4 and CCL5 and even higher amounts of IFNγ from human NK cells within 4 h. In this setup, glucose depletion and hypoxia, adverse conditions that characterize inflammatory tissue sites^11,12,62^, alone and in combination did not limit the early release of these cytokines from human NK cells which we concluded to be a metabolically autonomous NK cell response^34^. This glucose and oxygen independence of the cytokine response suggests that the inhibitory STAT3 effect on the cytokine response seen here (Fig. 4) was likely not predominantly due to the detrimental effect of STAT3 inhibition on glycolysis (Fig. 3F,G) but involved another downstream mechanism.

STAT3 suppresses expression of the cytotoxic effector molecules perforin and granzyme B^27^. Both these proteins are already integral components of the pre-existing cytotoxic granules, and the actual importance of STAT3 signaling for their immediate deployment upon contact of human NK cells with target cells, i.e., for degranulation, is not known and was, additionally, tested here. Because the mTORC1/mTORC2 inhibitor Torin2 was previously shown to reduce degranulation of mouse NK cells challanged with plate-bound anti-NK1.1 for 4 h^64^, we included the mTORC1 inhibitor rapamycin, besides the STAT3 inhibitor S3I-201, also in this experiment. Leukemic K-562 cells do not express surface major histocompatibility compatibility complex class Ia ligands for inhibitory natural killer cell receptors but high levels of ligands for NKG2D and the Natural Cytotoxicity Receptors^63^. These properties favor NK cells degranulation, and K-562 cells were thus chosen as target cells. Neither of the two compounds, however, significantly affected K-562 cell induced degranulation under normoxia and hypoxia (Fig. 5). Given the detrimental effect of STAT3 inhibition on glycolysis (Fig. 3F,G), this indicates that early degranulation does also not depend on this metabolic pathway.

In summary, the results presented here show that IL-15 priming synergizes with hypoxia in augmenting the HIF-1α response of human NK cells through STAT3 signaling. This response involves broad activation of glycolytic gene transcription but no changes in glycolytic proteins other than an elevation of cell surface GLUT1 that is likely responsible for a moderate increase in glycolysis in agreement with our previous study^34^. STAT3 is required for an effective early cytokine response upon interleukin treatment but not for leukemic target cells induced degranulation. Neither of these two processes apparently depends on glycolysis and normoxic oxygen levels in the early phase. However, target cell contact actually induces both these NK effector functions concomitantly, and it will be interesting to see whether cytokine release in this setting displays different metabolic requirements.

Future studies should address whether adaption to hypoxia through STAT3-HIF-1α signaling, as NK cells reach inflammatory sites and undergo priming, benefits later switching to aerobic glycolysis and thus effector functions. This could inform therapeutic strategies for combining adoptive NK cell transfer and IL-15 administration with targeting of STAT3 in cancer and attenuating the known interference of STAT3 inhibition with NK cell anti-tumor activity^51,52^. We propose that the observed detrimental effects of STAT3 inhibition on the HIF-1α, the metabolic and the cytokine responses in human NK cells contributes to this interference. The inhibitory effect of blocking STAT3 signaling on the early cytokine response but not on NK cell degranulation suggests that the delivery of STAT3 inhibitors may be beneficial only after IL-15 priming has been allowed to enhance NK cells adaption to the hypoxic tumor environment, e.g., in the context of a combination of NK cell and IL-15 therapy with STAT3 inhibition. Judging the actual importance of the HIF-1α response and of glycolysis and cellular respiration will require tumor models where hypoxia critically limits the NK cell dependent anti-tumor activity. Interestingly, Ni et al. (2020) have recently reported that NK cell specific conditional deletion of the HIF1A gene in mice enhances the anti-tumor activity of NK cells, presumably, up on their prolonged exposure to tumor hypoxia^65^. In this scenario, targeting of both mechanistically linked transcription factors, STAT3 and HIF-1α, may be of benefit.

## Supplementary Information

Supplementary Information

## Data Availability

The datasets used and/or analyzed during the current study are available from the corresponding author on reasonable request.
