# Supplementary material for: STAT3 governs the HIF-1α response in IL-15 primed human NK cells
Source: Sci Rep. 2021 Mar 29;11:7023. doi: 10.1038/s41598-021-84916-0 (PMC8007797; doi:10.1038/s41598-021-84916-0)
Supplement: Supplementary file 1 — Supplementary Information [file 41598_2021_84916_MOESM1_ESM.pdf]

Supplementary Material

STAT3 governs the HIF-1α response in IL-15 primed human NK cells

Anna Coulibaly, Sonia Y. Velásquez, Nina Kassner, Jutta Schulte, Maria Vittoria Barbarossa, Holger A. Lindner

Correspondence: Holger.Lindner@medma.uni-heidelberg.de

Contents

- Supplementary Figures S1–S8
- Western blot raw images for Figures 1B, 1D, 1E, 2B, 3E, S1 and S3
- Table S1

Figure S1

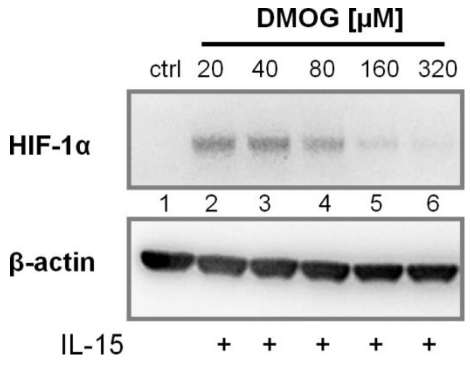

Fig. S1 Dimethyloxalylglycine (DMOG) was used to establish chemical hypoxia.

NK cells incubated for 16 h under standard conditions (ctrl, lane 1) were primed with IL-15 for 6 h in the presence of indicated DMOG concentrations (lanes 2–6) and subjected to immunoblot analysis with antibodies against HIF-1α and β-actin. HIF-1α did not further increase from 20 to 40 µM DMOG and decreased at higher concentrations.

Figure S2

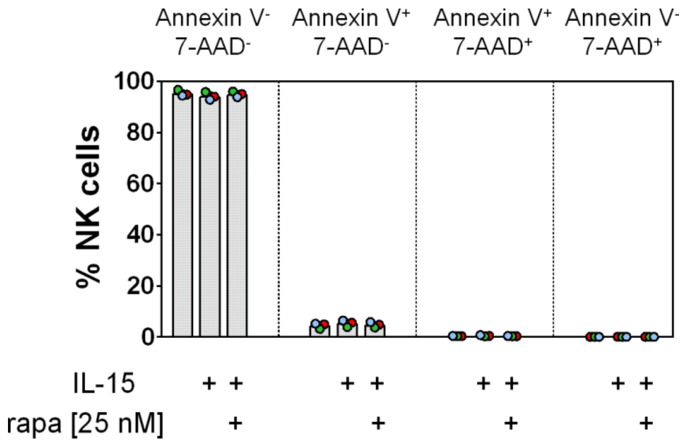

Fig. S2 Effect of rapamycin (rapa) on the viability of NK cells. Annexin-V and 7-AAD staining of unstimulated and IL-15 treated cells in the presence of 25 nM of rapa (n=3 ± SD). Single-positivity for annexin V and 7-AAD identifies early apoptosis and necrosis, respectively, and double-positivity late apoptosis.

Figure S3

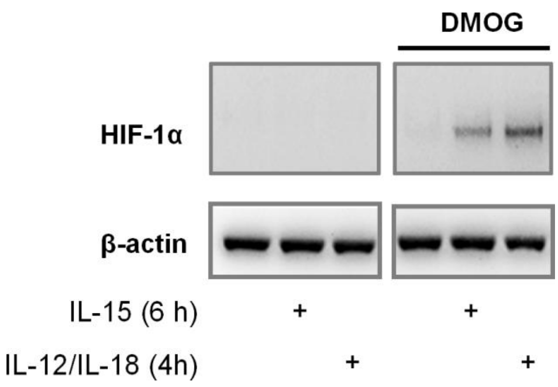

Fig. S3 IL-15 priming and combined stimulation with IL-12 and IL-18 both synergize with chemical hypoxia in inducing HIF-1α protein accumulation. NK cells were incubated with or without DMOG and cytokines for indicated times and subjected to immunoblot analysis with antibodies against HIF-1α and β-actin.

Figure S4

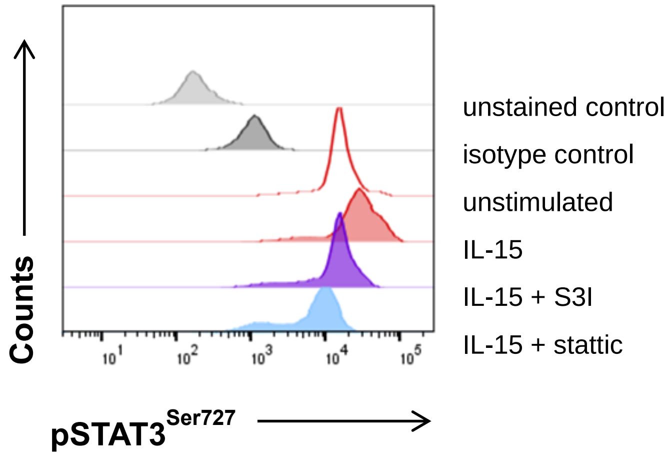

Fig. S4 Representative histogram for flow cytometric pSTAT3^{Ser727} determination corresponding to Fig. 3b and c in the main manuscript.

Figure S5

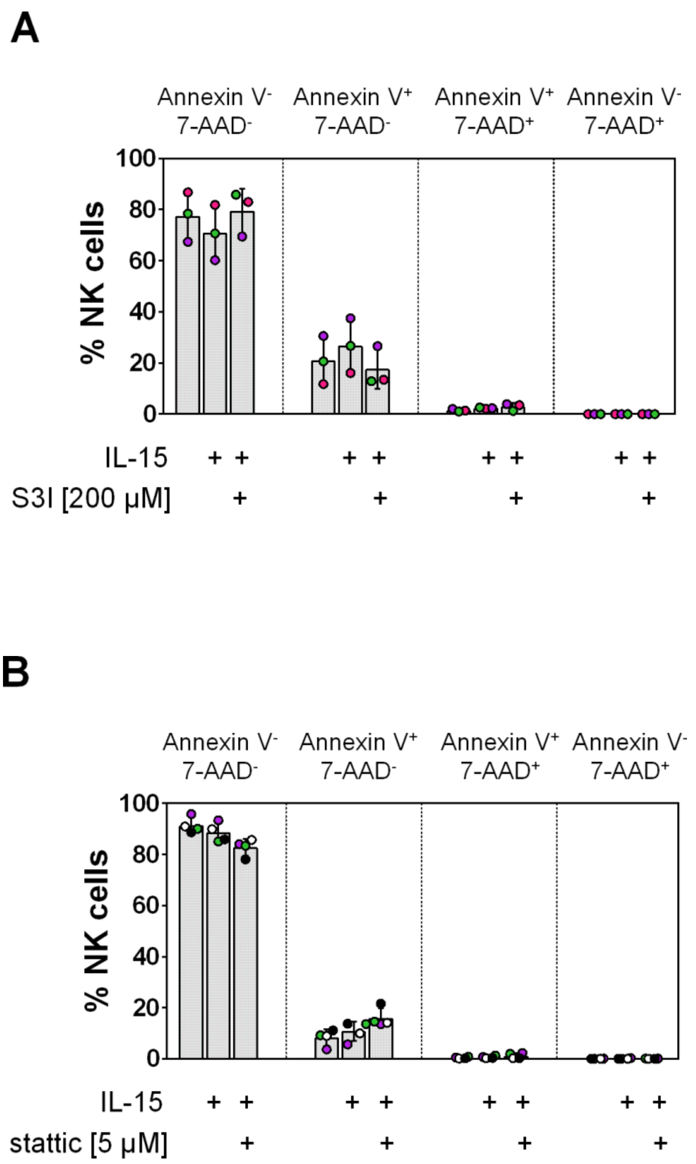

Fig. S5 Effects of STAT3 inhibitors on the viability of NK cells. Annexin-V and 7-AAD staining of unstimulated and IL-15 treated cells in the presence of **(a)** S3I-201 (S3I) and **(b)** stattic at indicated concentrations (n=3–4 ± SD). Single-positivity for annexin V and 7-AAD identifies early apoptosis and necrosis, respectively, and double-positivity late apoptosis.

Figure S6

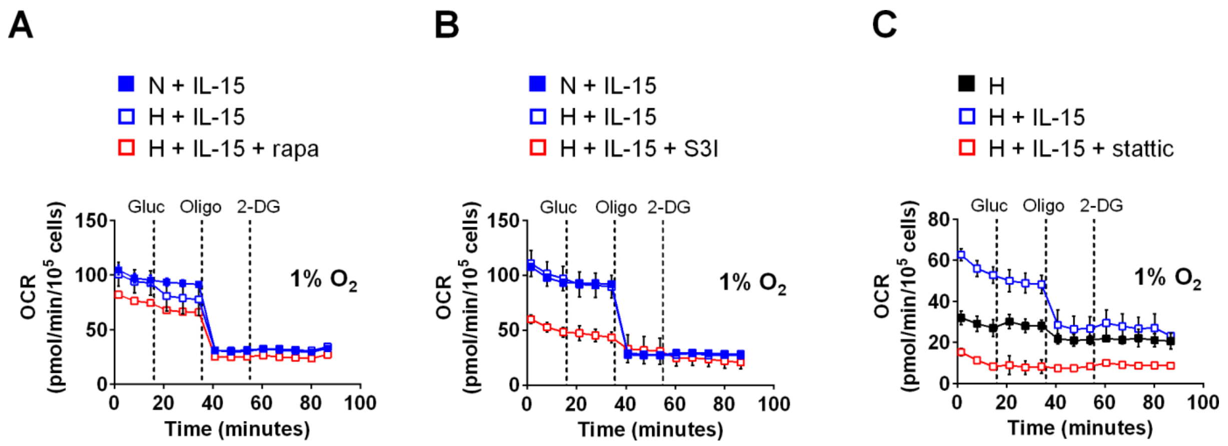

Fig. S6 Cellular respiration of NK cells at low oxygen during the glycolysis stress test. Cells were pre-cultured under normoxia (N) or hypoxia (H) and stimulated with IL-15 in the presence or absence of **(a)** rapamycin, **(b)** S3I-201 and **(c)** stattic. Oxygen consumption rate (OCR) values were measured over time in a hypoxic chamber at 1% O_2. Dashed lines indicate additions of glucose (Gluc), oligomycin (Oligo), and 2-deoxyglucose (2-DG). The OCR traces shown in **(a)**, **(b)** and **(c)** correspond to the simultaneously acquired ECAR traces shown in Fig. 2d and Fig. 3f and 3g in the main manuscript, respectively.

Figure S7

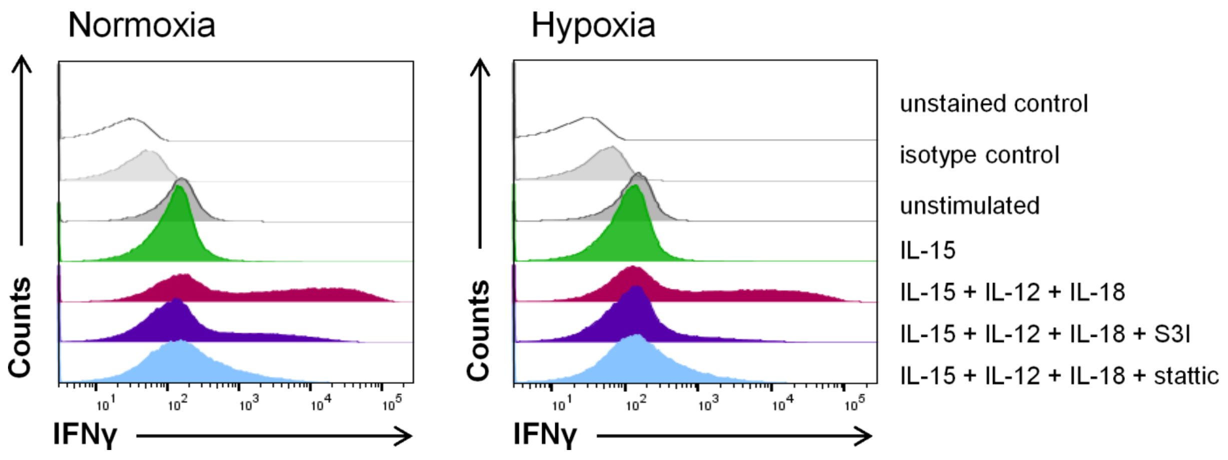

Fig. S7 STAT3 promotes IL-15/IL-12/IL-18 induced IFNγ production in NK cells. Representative histograms for flow cytometric IFNγ determination in normoxic and hypoxic NK cells corresponding to Fig. 4d in the main manuscript.

Figure S8

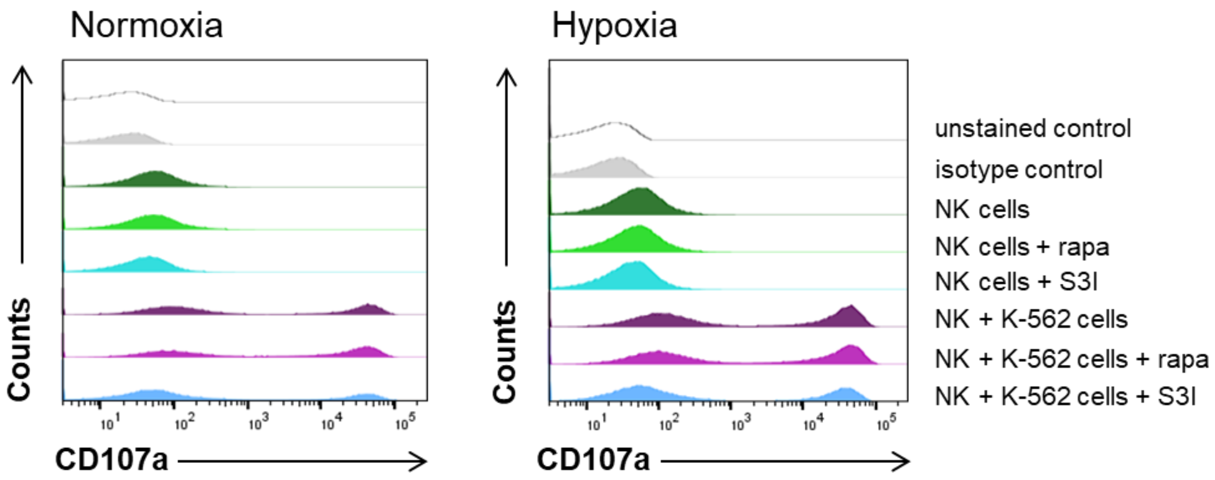

Fig. S8 Chemical mTORC1 and STAT3 pathway inhibition does not affect NK cell degranulation upon target cell contact. Representative histograms for flow cytometric determination of cell CD107a at the cell surface as a marker of degranulation in normoxic and hypoxic NK cells corresponding to Fig. 5 in the main manuscript. In addition to the co-incubation cultures, NK cells alone are included (histograms 3–5 from the top).

HIF-1α **β-actin**

Fig. 1b

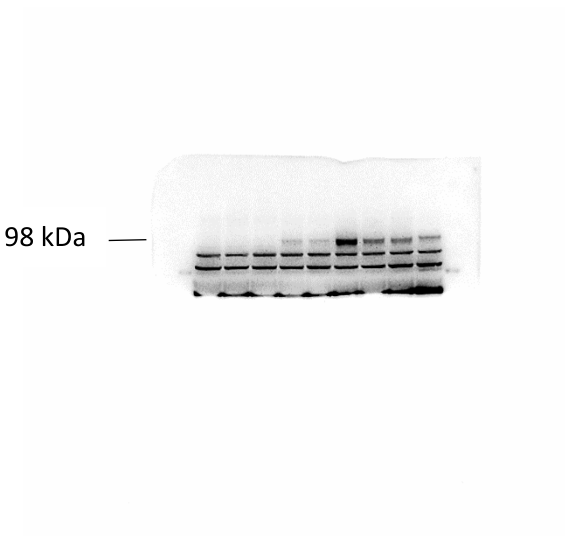

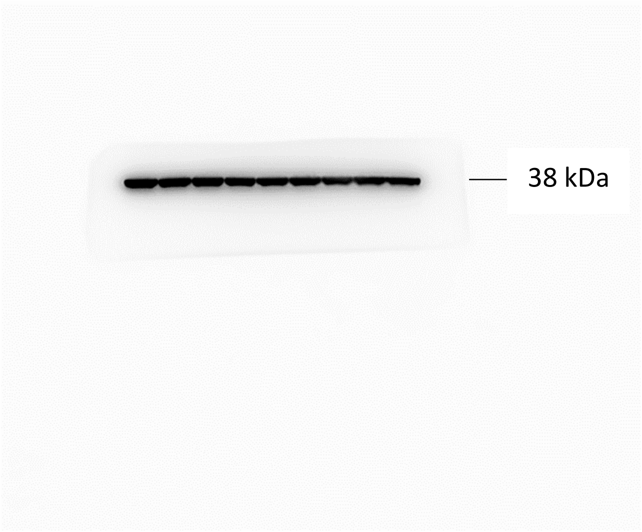

Fig. 1d

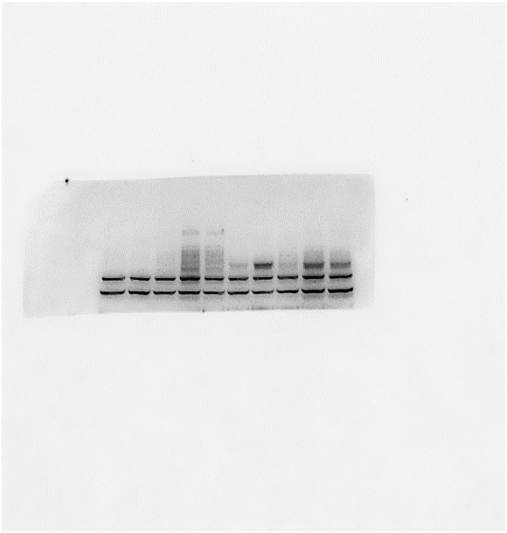

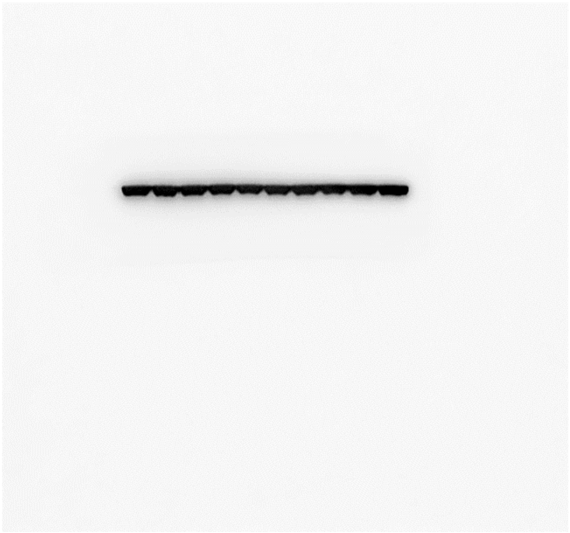

Fig. 1e – upper blot

HIF-1α

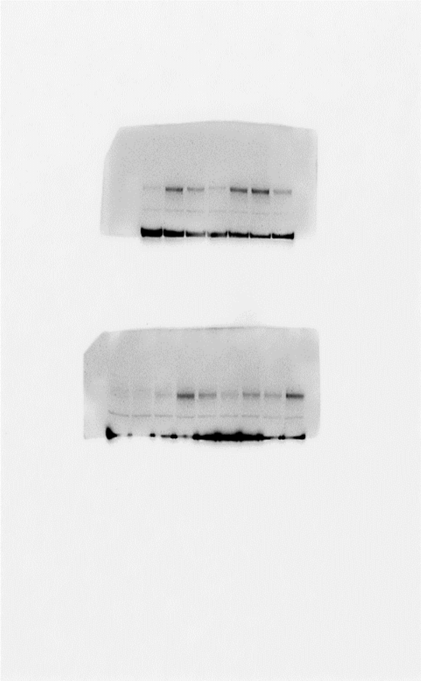

β-actin

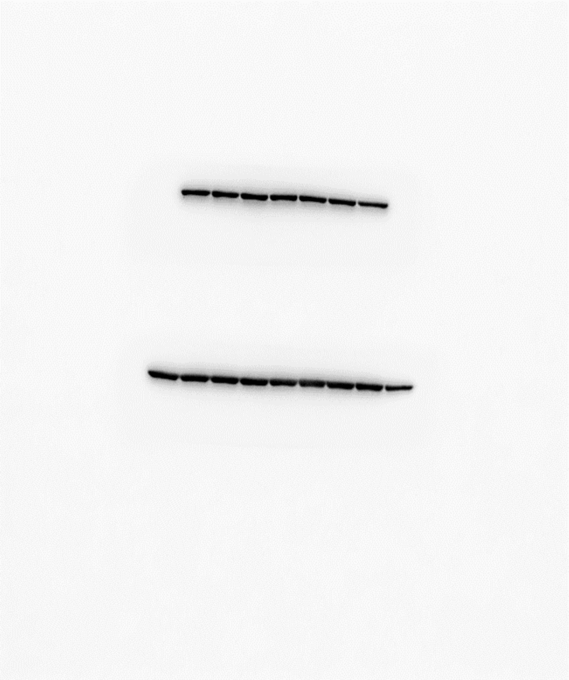

Fig. 2b

PDK1

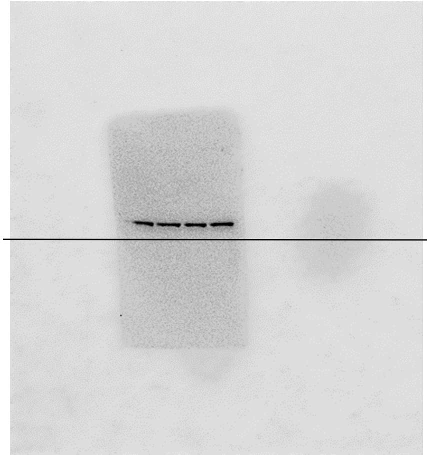

PGK1

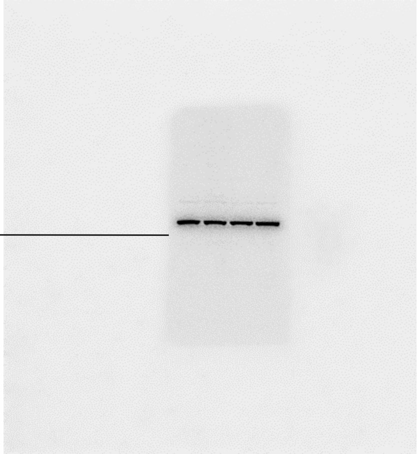

TPI

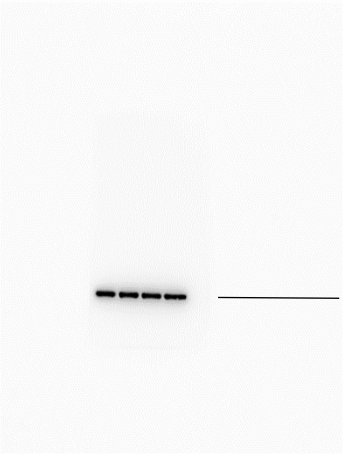

38 kDa

28 kDa

HIF-1α β-actin

Fig. 3e – lower blot

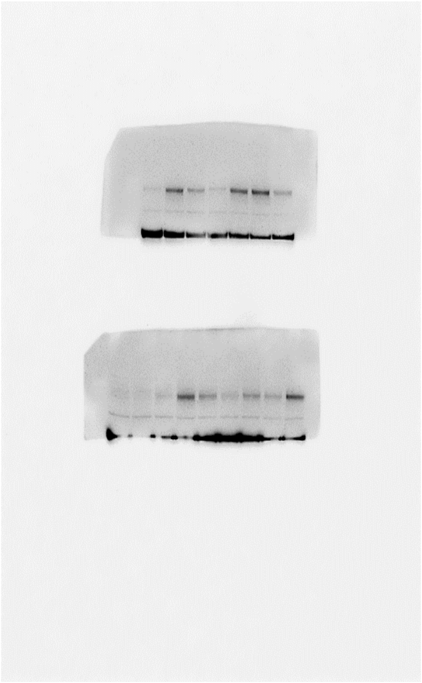

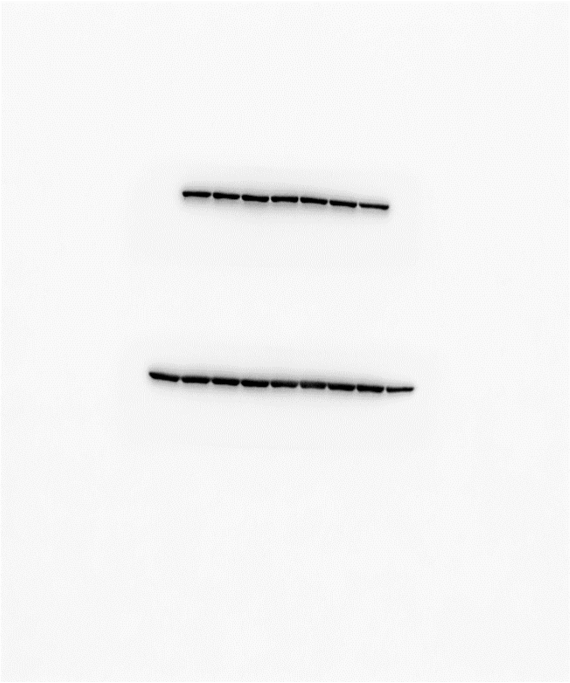

HIF-1α β-actin

Fig. S1

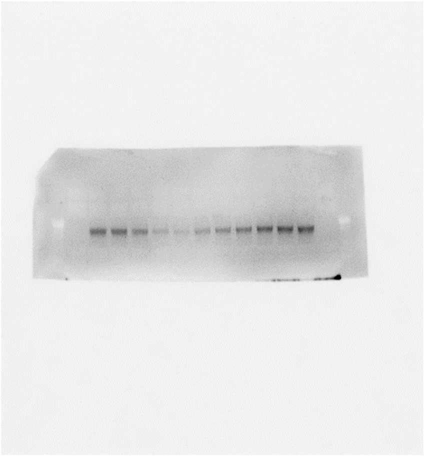

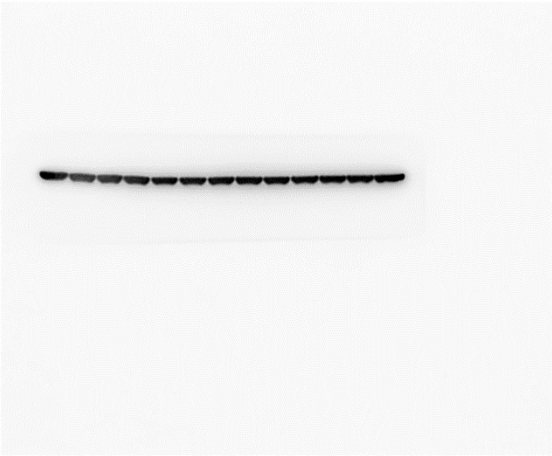

HIF-1α

β-actin

Fig. S3 – lower blot

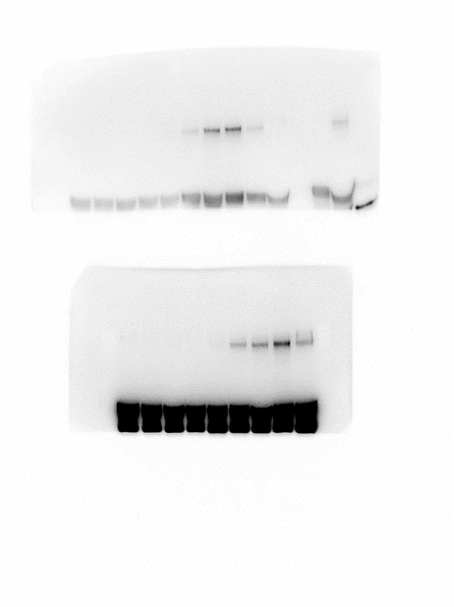

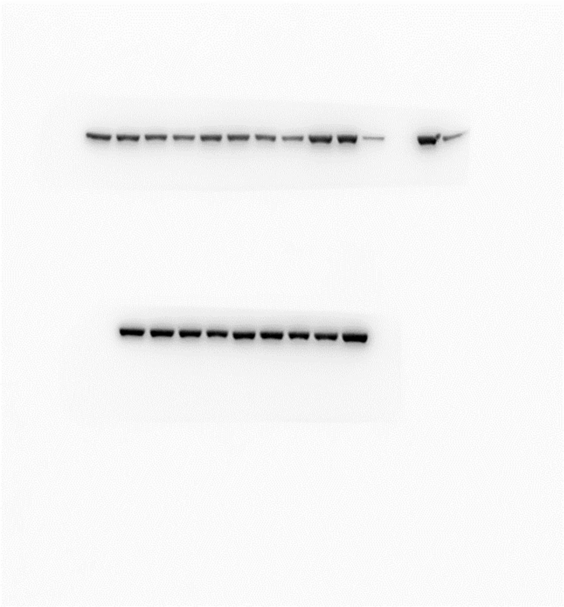

Table S1. Specifications of the TaqMan assays (Applied Biosciences) used for RT-PCR.

Protein	Gene	Assay ID
fructose-bisphosphate aldolase C	*ALDOC*	Hs00902799
BCL2/adenovirus E1B 19 kDa protein-interacting protein 3-like	*BNIP3*	Hs00969291
prolyl hydroxylase domain-containing protein 2 (PHD2)	*EGLN1*	Hs00254392
β-glucuronidase	*GUSB*	Hs00939627[a]
hypoxia inducible factor 1α	*HIF1A*	Hs00153153
pyruvate dehydrogenase kinase isozyme 1	*PDK1*[b]	Hs01561850
6-phosphofructo-2-kinase/fructose-2,6-biphosphatase 3	*PFKFB3*	Hs00998700
phosphoglycerate kinase 1	*PGK1*	Hs00943178
prolyl 4-hydroxylase subunit α-1	*P4HA1*	Hs00914594
pyruvate kinase muscle	*PKM*	Hs00761782
triosephosphate isomerase	*TPI1*	Hs03806547

[a] Endogenous control gene.

[b] Not to be confused with phosphoinositide-dependent kinase 1 protein.
